# Health Effects Associated With Electronic Cigarette Use: Automated Mining of Online Forums

**DOI:** 10.2196/15684

**Published:** 2020-01-03

**Authors:** My Hua, Shouq Sadah, Vagelis Hristidis, Prue Talbot

**Affiliations:** 1 University of California, Riverside Riverside, CA United States

**Keywords:** electronic cigarettes, vaping epidemic, vaping-associated pulmonary illness, e-cigarettes, electronic nicotine delivery devices, health effects, nicotine, symptoms, disorders, pulmonary disease, pneumonia, headaches, content analysis, text classification, e-cigarette, or vaping, product use associated lung injury

## Abstract

**Background:**

Our previous infodemiological study was performed by manually mining health-effect data associated with electronic cigarettes (ECs) from online forums. Manual mining is time consuming and limits the number of posts that can be retrieved.

**Objective:**

Our goal in this study was to automatically extract and analyze a large number (>41,000) of online forum posts related to the health effects associated with EC use between 2008 and 2015.

**Methods:**

Data were annotated with medical concepts from the Unified Medical Language System using a modified version of the MetaMap tool. Of over 1.4 million posts, 41,216 were used to analyze symptoms (undiagnosed conditions) and disorders (physician-diagnosed terminology) associated with EC use. For each post, sentiment (positive, negative, and neutral) was also assigned.

**Results:**

Symptom and disorder data were categorized into 12 organ systems or anatomical regions. Most posts on symptoms and disorders contained negative sentiment, and affected systems were similar across all years. Health effects were reported most often in the neurological, mouth and throat, and respiratory systems. The most frequently reported symptoms and disorders were headache (n=939), coughing (n=852), malaise (n=468), asthma (n=916), dehydration (n=803), and pharyngitis (n=565). In addition, users often reported linked symptoms (eg, coughing and headache).

**Conclusions:**

Online forums are a valuable repository of data that can be used to identify positive and negative health effects associated with EC use. By automating extraction of online information, we obtained more data than in our prior study, identified new symptoms and disorders associated with EC use, determined which systems are most frequently adversely affected, identified specific symptoms and disorders most commonly reported, and tracked health effects over 7 years.

## Introduction

### Background

At the time of their introduction 10 years ago, there was little information on the health effects associated with electronic cigarettes (ECs); nevertheless, they were often considered safer than conventional cigarettes because they do not burn tobacco and therefore produce aerosols with fewer chemicals. Since their introduction, a wide range of studies concerning the health effects associated with ECs have been conducted using various approaches that include online informatics and survey studies [1-6], short-term physiological assessments of EC use on human health [7,8], and *in vitro* and *in vivo* cytotoxicity studies [9-15]. Although these studies are limited mainly to acute exposures, they often suggest that EC use is not harm free [16]. Summaries of the health effect data and case report information on ECs can be found in 2 recent reviews [17,18].

Infodemiological approaches, which mine data from the internet and social media, have yielded new information such as EC topography and the effects of EC use on human health [1,19-22]. For example, in a previous study, we mined internet data on EC puffing topography and showed that puff duration is about twice as long for EC users than conventional smokers [22]. In addition, topography is highly variable among EC users, who generally intake much larger volumes of aerosol than cigarette smokers [23]. In our prior infodemiological study, we mined information manually from major EC online health forums and identified numerous negative and some positive health effects that users attributed to ECs [1]. This was a useful approach; however, manual mining methods are labor intensive, limit the number of posts that can be reasonably extracted and analyzed, and are not practical for examining large amounts of data over time.

### Objectives

The objective of this study was to use automated computer methods to mine an online forum and extract a large set of posts dealing with the effects of EC use on human health. These data were analyzed to identify the symptoms (undiagnosed conditions) and disorders (physician-diagnosed terminology) associated with EC use. Data were analyzed over a 7-year period, and the sentiment in each post (positive, negative, and neutral) was determined.

## Methods

### Datasets

**Figure 1:**
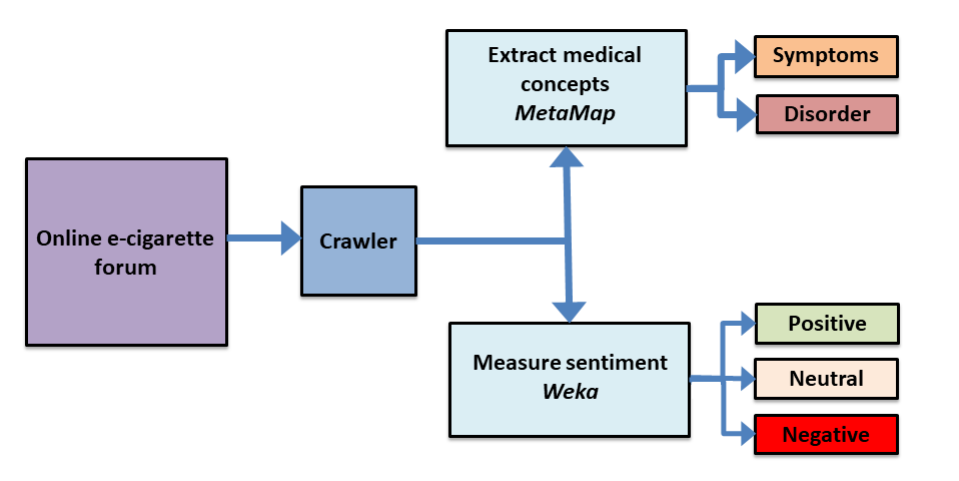
Online forum data pipeline showing processing, post sorting, and classification workflow.

We collected data posted between January 2008 and July 2015 on a large EC discussion forum. Data from 2008 and 2015 were each collected for approximately 6 months. We analyzed the layout of the website and built a crawler in Java using the Java library jsoup [24], which is designed to extract and parse information from HTML pages. The posts were collected from 7 subforums. The total number of discussion threads was 2330 and the total number of posts was 1,450,896. As the primary goal of this study was to evaluate the health effects produced by ECs, we focused on those posts that belonged to the 7 health subforums, which contained 41,216 posts. We emphasize that all collected data are publicly available, including discussion threads and users’ information. Figure 1 shows the overall pipeline used for our analysis.

### Medical Concepts

We used a modified version of the MetaMap tool [25] to annotate each post with a set of medical concepts from the Unified Medical Language System (UMLS). UMLS is a repository of a large number of biomedical controlled vocabularies [26]. In UMLS, there are 15 high-level semantic groups, which were created to help reduce complexity by grouping the semantic types [27]. In this work, we analyzed 2 semantic types, *sign or symptoms* and *disorder or syndrome*, which belong to the *Symptoms* and *Disorders* semantic groups. Each concept in UMLS can be assigned to multiple semantic types, but only to 1 semantic group [27]. As MetaMap was built to annotate the natural language text in biomedical academic publications, it is not very effective out-of-the-box on social media posts, as it successfully maps the medical terms most of the time, and not the descriptive or nonmedical terms [28]. To improve the tool’s mapping efficiency, we manually examined and removed misclassified UMLS concepts generated by MetaMap by performing the following steps:

1. For the 2 semantic types (symptoms and disorders), we ordered the concepts by their frequencies.
2. We analyzed the different terms mapped to each concept.
3. We removed the misclassified concepts from our results. Examples of misclassified concepts include:
  - mod, which refers to vape mods, was mapped to Type 2 diabetes mellitus (C0011860)
  - ect, which is a type of vape mod, was mapped to Benign Rolandic epilepsy (C2363129)
  - pic was mapped to Punctate inner choroidopathy (C0730321)

For each semantic type, we reported the most frequent disorders and symptoms overall and by year.

### Sentiment

To measure the positive and negative health effects produced by EC use, we used a supervised learning classifier (Random Forest) on a set of manually labeled posts to predict the sentiment for unseen posts. We randomly selected 1080 posts, which were labeled independently by 3 of the authors as the following:

- Negative: if a post clearly contained a health effect or unpleasant experience or complaint that co-occurred with the use of EC.
- Positive: if a post clearly mentioned a health improvement or a recovery from previous health effects when switching from smoking analogs to EC.
- Neutral: if a post did not express any sentiment.

Our interpretation of positive and negative is different from typical sentiment classifications, and mainly focuses on health-related effects. We first asked the labelers to categorize 400 posts, and then we measured the intercoder reliability between the labelers. Using *ReCal* [29], an online tool to calculate the reliability for the masses, the agreement was 80.53% using the *Average Pairwise Percent Agreement* measure. Owing to the high agreement, the rest of the posts were split evenly among the labelers to categorize. Table 1 shows the class distribution of our sample data with examples for each class; 44.7% (179/400) of posts were labeled as negative, 38.5% (154/400) as neutral, and 16.7% (67/400) as positive.

**Table 1:** Sample data summary.

Class	Posts, n	Example
Positive	180	“I’ve only been vaping for 2 1/2 weeks, but I’ve already noticed a big difference in my lungs (after 20+ years of smoking). For example, I had a chest cold when I started, and in the past, once a cold moved into my chest it took a couple of months to get rid of it. ...E-cigs are pretty darn amazing, IMHO.” [sic]
Neutral	416	“I dont [sic] think there are any tests since flavoring were not meant to be inhaled [sic]. I think we are taking our chances untill [sic] some evidence comes out...”
Negative	484	“Hi Everyone, I have been using e-cigarrette [sic] for the past 2 months and very disappointed [sic] that I have to stop, reason being my teeth, gums are sensitive and my tooth cracked yesterday, I have to have a crown fitted.8-o [sic]. I think that the nicotine is seriously not good for the mouth. My husband and work collegue [sic] have also reported sore gums, little sores in the mouth…”

Using Weka machine-learning toolkit v. 3.8.1 [30], we first filtered our sample data after many experiments using StringToWordVector class filter, which filters strings into N-grams using WordTokenizer class, with the following settings: (1) convert all words to lower case, (2) remove stop words, (3) stem words using Weka built-in stemmer, (4) keep only terms that appear at least twice, and (5) retain unigram, bigram, and trigram. We then split the sample data as follows: (1) 962 posts for the training test and (2) 118 posts for the test set. We then trained our data using the Random Forest classifier; however, the classifier’s initial accuracy was not satisfactory.

To improve the classifier’s accuracy, we needed to address a well-known issue in our sample data, which is the imbalanced class distribution [31]. The Positive class, as seen in Table 1, only covers 16.7% (67/400) of the data, whereas the Neutral class covers 38.50% (154/400) and the Negative class covers 44.7% (179/400). Thus, we oversampled the Positive class by duplicating the posts which were labeled Positive in the training set only. Table 2 shows the new class distribution for the training set, namely Training (extended). Another approach we used to improve the accuracy is annotating all the posts in the sample data with the ancestors of the medical concepts mentioned in the posts. For example, if *pneumonia* is mentioned in a post, then we append with *Disorder of lung*.

After using the new training set, the classifier’s accuracy increased from 66.95% to 75.42%. Table 3 reports for each class 3 different measures, including precision, recall, and F-measure. As seen in the table, the classifier is most accurate on the Negative class (F-measure=0.79), followed by Positive and Neutral classes.

**Table 2:** Training data summary (N=400).

Class	Training, n (%)	Training (extended), n (%)
Positive	67 (16.75)	112 (28.0)
Neutral	154 (38.50)	136 (34.0)
Negative	179 (44.75)	152 (38.0)

**Table 3:** Test data classification accuracy (N=118).

Class	Precision	Recall	F-measure	Posts, n
Positive	0.73	0.72	0.74	21
Neutral	0.67	0.77	0.71	39
Negative	0.84	0.74	0.79	58
Average	0.76	0.75	0.76	118

### Data Categorization and Analysis

All health-related effects (symptoms and disorders) data reported by EC users in posts were collected iteratively and sorted into Microsoft Excel spreadsheets. The symptoms and disorders were further grouped according to the organ system and anatomical region, which we defined as *systems* previously [1]. When a symptom could have been associated with more than 1 system, the health effect was assigned to the system for which it had the strongest fit (eg, improved sense of taste was assigned to sensory but could have been mouth/throat). Frequency distributions for the overall grouped data in each system for symptoms and disorders were plotted using GraphPad Prism (GraphPad, San Diego). In addition, the sentiment for each post was grouped according to their positive, neutral, and negative sentiment as described in the Methods section.

## Results

### Overall Frequency of Reported Symptoms and Disorders Classified by System or Anatomical Region

**Figure 2:**
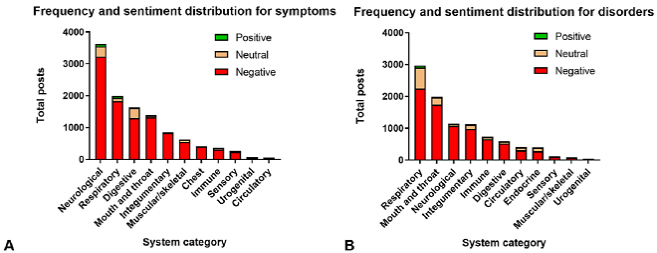
Frequency distribution of reported symptom (A) and disorder (B) posts grouped into their systems or anatomical regions. The frequency of positive, neutral, and negative posts is shown for symptoms (A) and for disorders (B).

The 41,216 posts we collected spanned the years from 2008 to 2015 (2008 and 2015 were half years). We analyzed the frequency of reports for various symptoms and disorders by consolidating the reported health effects into structural or physiological systems (eg, sore throat was classified into mouth and throat; Figure 2). The 5 systems that had the most reports for symptoms were neurological (n=3623), respiratory (n=1995), digestive (n=1637), mouth and throat (n=1390), and integumentary (n=853; Figure 2). The top 5 systems for disorders were respiratory (n=2972), mouth and throat (n=1986), neurological (n=1143), integumentary (n=1123), and immune (n=739; Figure 2). For both symptoms and disorders, a majority of the posts were associated with negative sentiment across all systems (Figure 2).

### Symptom and Disorder Frequency and Sentiment Distribution Over Time

**Figure 3:**
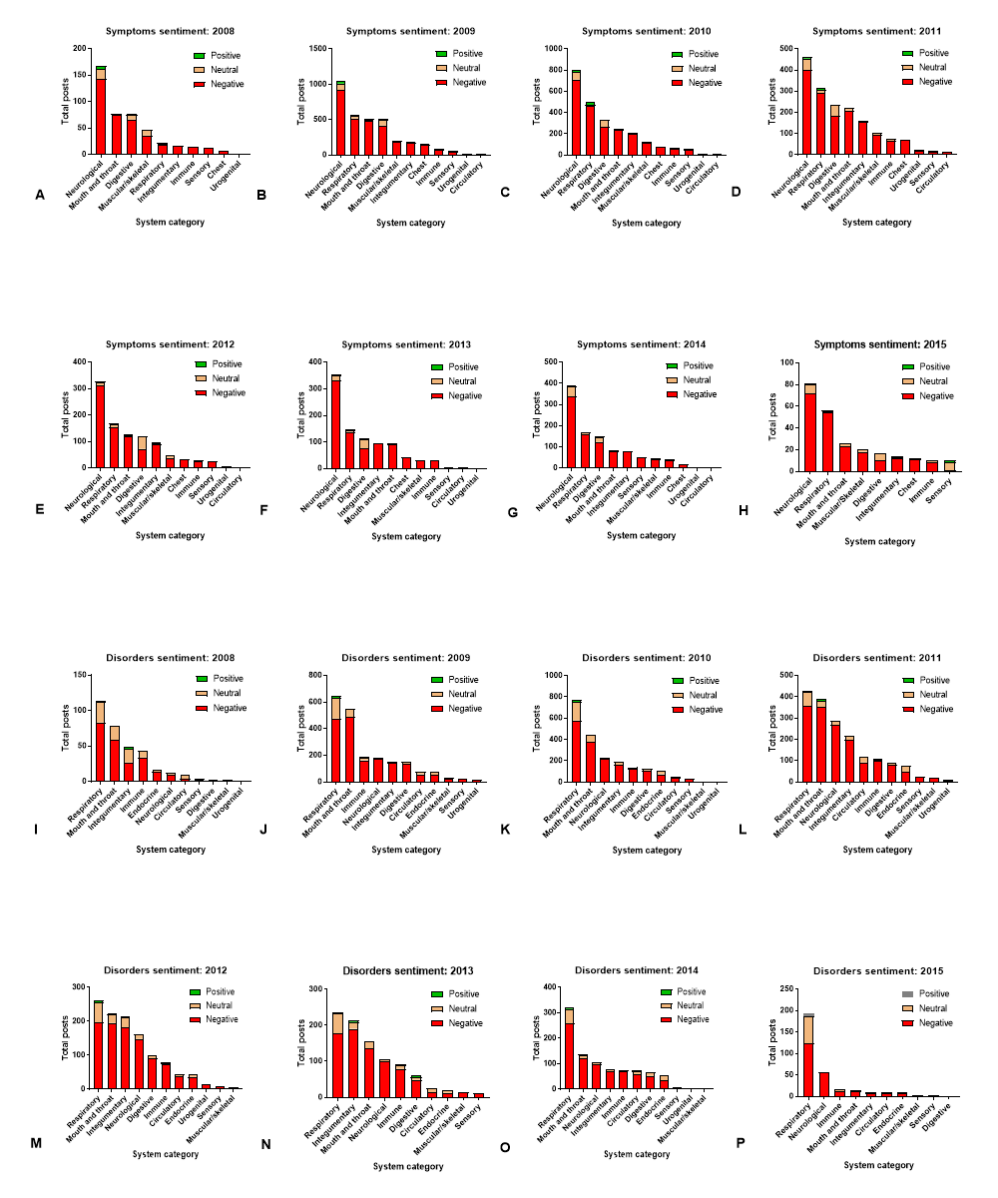
The frequency distribution of positive, neutral, and negative sentiment was assigned for reported symptoms (A-H) and disorders (I-P).

After examining overall frequency distribution for all posts, we grouped the posts according to their years for analysis in their symptom or disorder categories. Across all years for both symptoms and disorders, we found the frequency distribution of reports per year. In addition, the posts for symptoms and disorders were categorized according to sentiment (positive, negative, and neutral), and their frequency per year was summarized in stacked bar graphs for each year (Figure 3).

For the symptoms, the posts with the most reports were consistently found in the neurological, respiratory, digestive, integumentary, and mouth and throat systems. For all years except 2008, the neurological and respiratory systems were the top 2 systems. The digestive, integumentary, and mouth and throat alternated in some years, but were generally in the top 5 systems with the most posts in each of the years.

Similarly, the posts containing disorders associated with EC use had similar results for their top 5 system categories across the 7 years of reporting. The 2 top systems reported between 2008 and 2012 were the respiratory and mouth/throat. Alternating in the top 5 disorders were the integumentary, neurological, and immune systems.

Negative sentiment was associated with most symptoms and disorders in each system or anatomical region (Figure 3), with some increases reported in positive health effects in 2015 for the disorders (Figure 3). It should also be noted that we only have partial reporting for 2015 because data collection was terminated by the EC forum.

### Specific Symptoms and Disorders in Systems With the Most Reports

**Figure 4:**
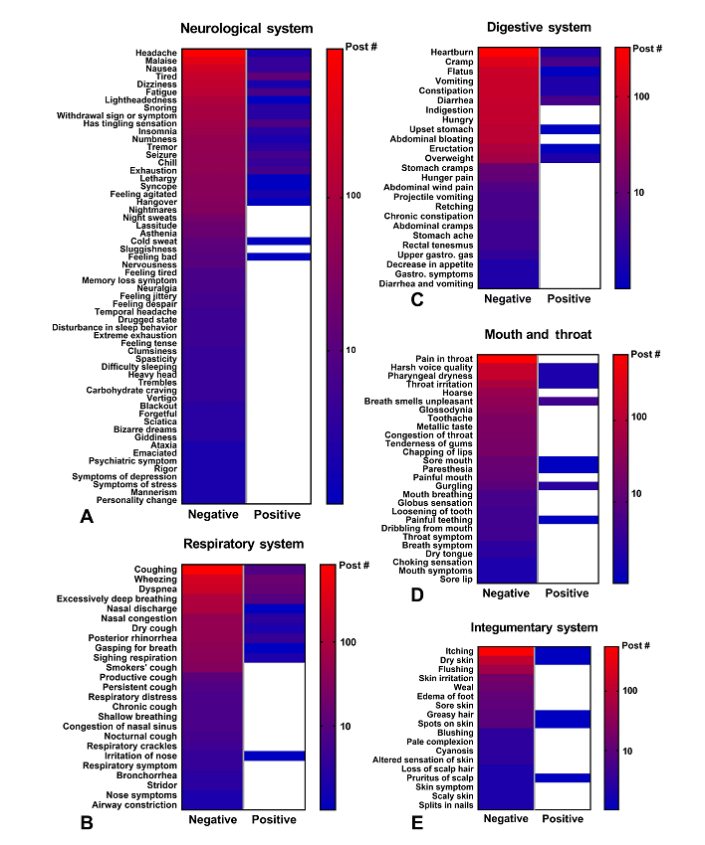
Heat map of specific symptoms reported in the neurological, respiratory, digestive, mouth and throat, and integumentary systems. The total number of posts for each symptom is shown on a log scale ranging from high (red) to low (blue).

**Figure 5:**
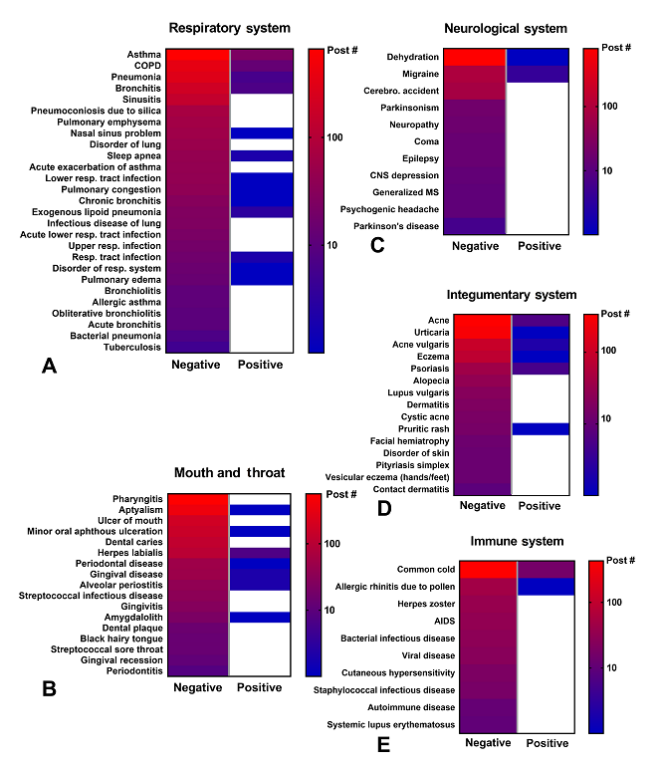
Heat map of specific disorders reported in the respiratory, mouth and throat, neurological, integumentary, and immune systems. The total number of posts for each disorder is shown on a log scale ranging from high (red) to low (blue).

Heat maps were made by plotting the frequency with which individual symptoms/disorders occurred for all 41,216 posts (Figures 4 and 5; Multimedia Appendices 1 and 2). Symptoms with fewer than 2 posts are listed in Multimedia Appendix 3. The total number of negative and positive posts for each symptom or disorder is shown on a log scale ranging from high (red) to low (blue). White represents a zero-post frequency. Numerous negative symptoms were reported for each system. Typically, about 16.52% (6807/41,216) of the symptoms were reported frequently (red), while the majority often occurred in fewer than 100 posts (blue to purple). In the neurological system, the most common symptoms included: headaches (n=939), fatigue/tired/malaise (n=468), nausea (n=290), dizziness (n=183), and lightheadedness (n=113; Figure 4). In the respiratory system, the negative effects included: coughing (n=852), wheezing (n=298), dyspnea (n=235), and excessively deep breathing (n=112). The most reported symptoms in the digestive system were: heartburn (n=327), cramping (n=303), flatus (n=176), and constipation (n=113). In the mouth/throat and integumentary systems, common symptoms were: pain in throat (n=643), harsh voice quality (n=175), pharyngeal dryness (n=147), itching skin (n=565), and dry skin (n=121; Figure 4). Other commonly reported symptoms involved aching and chest pains as well as immune symptoms related to the cold and flu.

Although positive symptoms were not frequently reported in this online forum, those reports that were posted most often dealt with improvements in the neurological (n=77), respiratory (n=60), digestive (n=19), and mouth and throat (n=18) systems (Figure 4). In the neurological system, these include improvement in tiredness (n=12) and insomnia (n=8). For respiratory system, these symptoms included improvements in wheezing (n=17), dyspnea (n=14), and coughing (n=8). In the digestive and mouth and throat systems, improvements were found in cramp (n=5) and halitosis (n=5). Other systems and anatomical regions had fewer than 10 total positive reports.

For each system/anatomical region, there were 1 to 3 top disorders. In the respiratory system, the most common disorders were asthma (n=916), chronic obstructive pulmonary disorder (COPD; n=471), pneumonia (n=367), and bronchitis (n=232; Figure 5). In mouth and throat, the most common disorders were pharyngitis (n=565), aptyalism (n=377), and ulcer of mouth (n=207). The most reported disorders in the neurological system were dehydration (n=403) and migraine (n=103). Most disorders were reported in the respiratory, mouth and throat, neurological, integumentary, and immune systems (Figure 5), whereas the remaining systems had fewer reported disorders (Multimedia Appendix 2).

**Figure 6:**
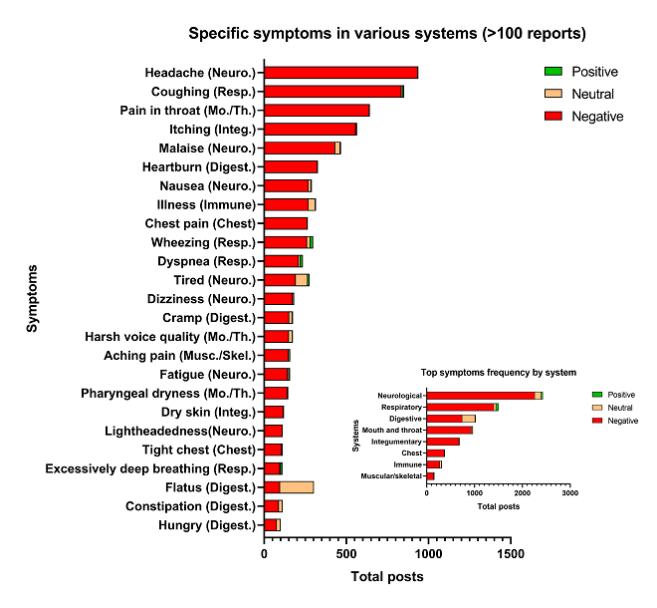
Frequency distribution of specific symptoms with over 100 posts and frequency distribution of their systems or anatomical regions (inset). Digest.: digestive; Integ.: integumentary; Mo./Th.: mouth and throat; Musc./Skel.: muscular/skeletal; Neuro.: neurological; Resp.: respiratory.

**Figure 7:**
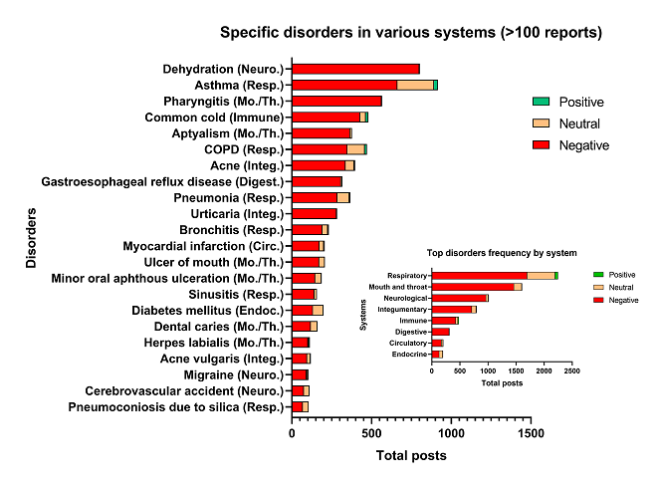
Frequency distribution of specific disorders with over 100 posts and frequency distribution of their systems or anatomical regions (inset). Neuro.: neurological; Resp.: respiratory; Mo./Th.: mouth and throat; Integ.: integumentary; Digest.: digestive; Circ.: circulatory; Endoc.: endocrine.

To compare the frequency with which different symptoms/disorders appeared across different systems, frequency distribution graphs were created (Figures 6 and 7). Graphs show only those symptoms/disorders with over 100 posts (Figures 6 and 7). These data were sorted by negative sentiment as negative effects were most commonly reported and were of most interest. In total, 25 symptoms and 22 disorders had over 100 posts. The 5 top symptoms in the 41,216 posts were: headache, coughing, pain in throat, itching, and malaise (Figure 6). The top 5 disorders in the dataset were dehydration, asthma, pharyngitis, common cold, and aptyalism (Figure 7). These symptoms and disorders are the most commonly reported conditions in our dataset.

### Identification of Frequently Reported Paired Symptoms

**Figure 8:**
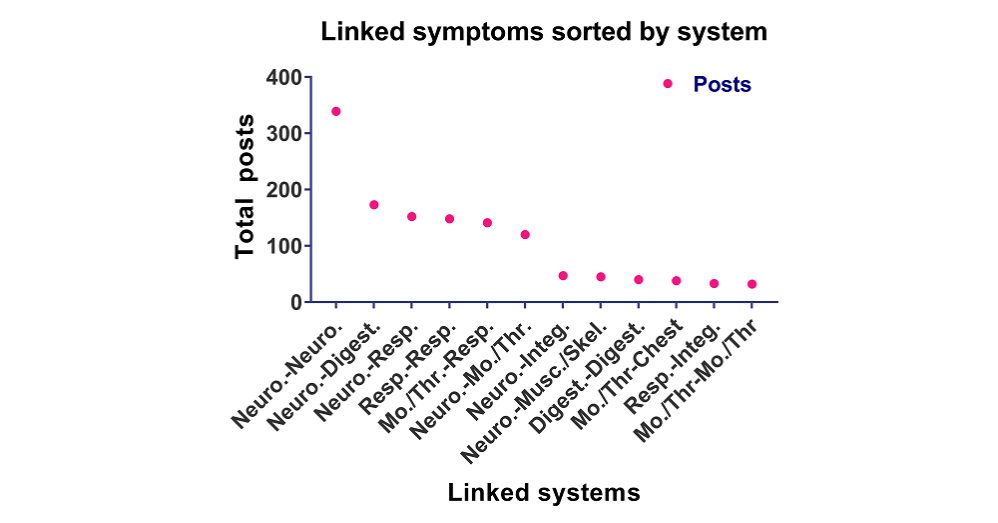
Graph showing frequency with which symptoms in various systems were linked. Digest.: digestive; Integ.: integumentary; Mo./Th.: mouth and throat; Musc./Skel.: muscular/skeletal; Neuro.: neurological; Resp.: respiratory.

A total of 46 paired symptoms were frequently reported (Figure 8; Multimedia Appendix 4). Those with over 30 reports included a combination of neurological-neurological symptoms (eg, nausea and headache), respiratory-respiratory symptoms (eg, wheezing and coughing), and/or neurological-respiratory-mouth and throat symptoms (eg, pain in throat and headache; coughing and headache). The results in the top symptom pairings reflect the abundance of symptoms reported in their respective categories. As for those pairings occurring in less than 30 posts, various combinations of neurological, respiratory, integumentary, and digestive related symptoms.

## Discussion

### Principal Findings

The internet is a dynamic resource containing information that can be mined to learn about the health effects associated with EC use. In our previous study, we manually mined 632 posts from 3 online EC forums to identify both positive and negative health effects reported by EC users [1]. Manual mining of such information is time consuming, labor intense, and limited by the number of posts that can be realistically evaluated. To take additional advantage of the internet as a repository of EC-related health information, we developed an automated method that was used to extract over 1 million posts from an EC forum. These posts were then filtered to yield over 41,000 health-related posts for detailed analysis. By automating the extraction process, we collected 100 times more posts for analysis and tracked responses over 7 years, an option that would be too time consuming to perform manually. The data showed a variety of positive and negative symptoms/disorders, demonstrating that the internet is a valuable resource for acquiring new data related to EC usage and their associated health effects.

The results from this study are in overall good agreement with our prior publication [1]. In both studies, the neurological and respiratory systems were most often reported to have adverse effects associated with EC use. Both studies reported similar positive and negative health effects in various systems, and within all systems, a small number of self-reported symptoms and disorders occurred at high frequency (Figures 6 and 7). However, with the power of automated mining, we were also able to (1) identify numerous symptoms and disorders, many of which were not previously reported, along with their frequencies; (2) report new data in the disorders category for each system; (3) identify those symptoms/disorders that users have reported most frequently which will be of interest to health care providers treating patients using EC products; (4) show that health effects were similar over a 7-year period; (5) evaluate positive and negative sentiments for symptoms and disorders; (6) evaluate symptoms that are linked to each other; and (7) identify top symptoms (eg, wheezing) and disorders (eg, asthma, COPD, and pharyngitis) that are associated with inflammation. In addition, some symptoms (eg, headache and nausea) and disorders (eg, pneumonia) that occurred with the highest frequency in the neurological and respiratory system have also appeared in a number of case reports in the EC literature [17,32-35]. Also, in agreement with our prior study, some reported health outcomes attributed to EC use were positive [1]. These included reduction in symptoms such as coughing and wheezing and disorders such as asthma, COPD, and common cold. It is very likely that there were real health benefits for some individuals, especially for those switching from conventional to EC use, and this is supported by other publications [36-38].

There are numerous reports on the health effects of EC, many of which are in agreement with our data. In the neurological system, the most commonly reported adverse symptoms we observed included headache, fatigue, nausea, dizziness, and seizures, which have also been reported in human studies [39-42]. Headaches have been reported to the Food and Drug Administration (FDA) by EC users [43], they were a common side effect in various surveys and online studies [4,44-46] and were reported in human studies in which participants used different EC devices and refill fluids with varying nicotine concentrations [47-49]. In 1 case report, an adult male experienced severe headaches/migraines and seizures for 1 week before being diagnosed with reversible cerebral vasoconstriction syndrome related to EC use [32], and in a second case, an adolescent female developed persistent daily headaches after a single EC use [50]. Nausea and dehydration were commonly reported symptoms and disorders of the neurological system. These symptoms have been associated with headache and fatigue/tiredness [51-54]. In our study, headache and nausea were frequently reported together, demonstrating that symptoms associated with EC use may be linked. The frequency distribution showing symptoms and disorders with over 100 posts also revealed that digestive symptoms such as heartburn and circulatory disorders such as myocardial infarction were highly reported. Digestive symptoms related to EC use have not been previously focused on and may be important to pay attention to, and disorders such as myocardial infarction associated with EC have recently received more attention from epidemiology studies [55].

For the respiratory system, the most frequently reported symptoms included coughing, wheezing, and dyspnea, and the top paired respiratory symptoms were coughing-wheezing. In the national Population Assessment of Tobacco Health (PATH) and in some human surveys, EC use was associated with increased wheezing (an important potential risk factor for respiratory disease) [56-59]. In our study, the top disorders were asthma, COPD, pneumonia, bronchitis, and sinusitis, which have a common theme of inflammation. Human studies and surveys have shown that adolescents and adults associated chronic bronchitis symptoms (eg, cough, phlegm, or dyspnea) with EC use [57,58]. Epidemiological studies have linked EC use to both COPD and asthma [56], and the PATH study showed that dual use of EC and conventional cigarettes aggravated this risk [56]. Frequently reported respiratory disorders in our study such as pneumonia and bronchitis have appeared in several EC case reports, most of which deal with lung inflammation and pneumonia-linked incidents [34,35,60,61]. Some of the patients in these case reports had no preexisting health conditions but presented with coughing, wheezing, and dyspnea. They typically recovered from their respiratory disorders after discontinuing EC use.

The circulatory, mouth/throat, chest, integumentary, and immunological systems were also affected by EC use in our study. Symptoms such as pain in throat, dry skin, pounding heart, and chest pain have been reported in survey/human EC studies [62]. Myocardial infarction, which was a top disorder for the circulatory system in our data, has been described in a case report after the patient used an EC with high nicotine [63]. A link between EC use and myocardial infarction was also found in a recent national survey adjusted for conventional smoking and other risk factors [55]. EC users are potentially susceptible to periodontal disease and increased plaque formation, which could lead to dental caries (also reported in our paper) [64]. Other reported disorders in our study, such as common cold and diabetes mellitus, are immunologically based, and multiple studies have shown EC aerosol exposure can induce inflammatory response [65]. Mice exposed to EC aerosol have impaired pulmonary viral and bacterial clearance ability that can lead to increased bacterial resistance, implying that EC users are more susceptible to cold and flu, a common complaint in our online studies. Experimental studies have further demonstrated that EC aerosol exposure can result in oxidative stress [13,66,67], suggesting chronic use could trigger inflammation, leading to progressive inflammatory disorders in the respiratory system.

### Electronic Cigarette Aerosol Chemicals That May Produce Adverse Health Effects

EC refill fluids and aerosols are complex mixtures that contain flavor chemicals, solvents, nicotine, and metals that could contribute to adverse health effects (Table 4). Although most flavor chemicals in EC are generally regarded as safe (GRAS) for ingestion, their inhalation safety has usually not been established [68]. Some EC products contain high concentrations of flavor chemicals that exceed the National Institute of Occupational Safety and Health limits [69-75] and the concentrations normally used in consumer products [71,72]. Many EC flavor chemicals are classified as irritants [72] and are cytotoxic when tested *in vitro* at concentrations below those in EC products [71,72]. Cinnamaldehyde, which is used in refill fluids including those that do not have cinnamon in their name [10], is highly cytotoxic in the 3-(4,5-dimethylthiazol-2-yl) -2,5-diphenyltetrazolium bromide [10,69] and impaired respiratory response in immunological assays [76-78] and *in vivo* assays. In cell, animal, and human studies, EC flavor chemicals (eg, citrus/fruit and chocolate) caused an increase in reactive oxygen species leading to tissue and DNA damage and inflammation [67], which could in turn lead to mutations and disease progression. Some flavor chemicals, such as alcohols and phenols, can dilate blood vessels and cause headache, nausea, and fatigue. Prolonged inhalation of flavor chemicals, such as benzaldehyde, ethyl butanoate, diacetyl and its derivatives (2,3-pentadione, acetoin), triacetin, and limonene can elicit headaches, dizziness, and/or respiratory symptoms. Diacetyl (2,3-butanedione), a diketone associated with respiratory symptoms (wheezing and shortness of breath) and bronchiolitis obliterans, is in some EC refill fluids and can form as a reaction product during aerosolization [70,74]. Additional reaction products that form in EC aerosols (eg, aldehydes, acetals, and oxides) can be harmful to humans and elicit various symptoms, including pain.

**Table 4:** Examples of chemical components in electronic cigarettes that may cause major symptoms/disorders with reference citations of studies.

Symptom/disorder	System	Flavor chemicals (study)	Metals (study)	PG^a^/VG^b^/byproducts (study)	Nicotine (study)
Headache	Neurological	[79,80]	[81-83]	[51,52,84,85]	[86-88]
Fatigue/malaise	Neurological	[89]	[90]	—^c^	—
Dizziness	Neurological	[91]	[92,93]	—	[94]
Nausea	Neurological	[79,80]	[92]	[85,95]	[87,96]
Dehydration	Neurological	—	—	[52,84]	[97]
Coughing	Respiratory	[91,98-100]	[62]	[101,102]	—
Wheezing	Respiratory	[57,99]	[103]	[104]	—
Dyspnea	Respiratory	[99]	[105,106]	[107]	[108]
Asthma	Respiratory	[99,109]	[92,103,104]	[110]	—
COPD^d^	Respiratory	[111]	[92]	[110]	—
Pneumonia	Respiratory	—	[103]	—	—
Bronchitis	Respiratory	[111,112]	[113]	—	—
Sinusitis	Respiratory	[79,114]	[115]	[114]	—
Pain in throat	Mouth and throat	—	—	[101]	[87]
Dental caries	Mouth and throat	[116]	[117]	—	[118]
Itching/urticaria	Integumentary	—	[119-121]	[122]	[123]
Dry skin	Integumentary	—	—	[124]	—
Acne	Integumentary	—	[125]	—	[126,127]
Heartburn	Digestive	—	—	—	[128]
Cramp	Digestive	—	[129]	—	—

^a^PG: propylene glycol.

^b^VG: vegetable glycerin.

**^c^**Lack of evidence in referenced literature.

^d^COPD: chronic obstructive pulmonary disorder.

Elements/metals (eg, aluminum, copper, cadmium, chromium, iron, nickel, silicon, lead, cobalt, and zinc) have been identified in EC aerosols [130,131]. In studies not involving ECs, these elements/metals have been linked to neurological (headache, nausea, and dizziness) and respiratory (eg, coughing, wheezing, shortness of breath, and bronchial/pulmonary irritations impairment) symptoms [132]. A positive correlation has been reported between human EC use and internal concentrations of nickel and chromium [133]. We found *pneumoconiosis due to silica* frequently reported by users, which could be caused by silica particles in EC aerosols [130]. Inhalation of silicon particles can elicit cough, inflammation, and lung fibrosis [134]. Although there is not a consensus on whether element/metal concentrations in EC aerosols are high enough to produce these effects [135], some evidence suggests that they could be a factor [130,136]. A female patient with no history of allergic disease tested positive for nickel allergy after being diagnosed with dermatitis caused by corrosion of the EC device [119]. Some of the top symptoms and disorders in our study also relate to inflammation of the skin (eg, itching and eczema), and this may be attributed to direct exposure and allergic reactions to EC products [119,120].

Propylene glycol and glycerin, 2 solvents in EC aerosols, are generally considered safe for ingestion; however, they are known respiratory tract and integumentary irritants [137]. When heated, propylene glycol can produce toxic aldehyde reaction products, such as acetaldehyde and formaldehyde [138], which can cause cellular and tissue damage in the body [67]. Inhalation of aldehyde fumes can cause dizziness, nausea, and headaches in humans [113], and formaldehyde can cause coughing, wheezing, pneumonia, bronchitis, and neurological and cardiovascular symptoms (eg, headaches, nausea, heart palpitations) [139]. Inhalation of propylene glycol mists can elicit both neurological and respiratory symptoms, such as nausea, wheezing, shortness of breath, and cough [101,140] and can exacerbate asthma [101,140]. Propylene glycol and glycerin produce 15 different aerobic thermal degradation products through hydrogen abstraction, oxidation, and cleavage reactions [138]. Several of these (eg, formaldehyde, formic acid, acetaldehyde, and acrolein) are carcinogens or have genotoxic potential [141,142]. Some of these byproducts are hemiacetals (such as formic acid and formaldehyde), which equilibrate and persist in the aerosols inhaled by the users.

Nicotine, a major component in most EC fluids, has various neurological, respiratory, digestive, mouth/throat, and circulatory system effects that overlap the symptoms/disorders observed in our study. Most cases of EC nicotine poisonings result from oral ingestion or intravenous injection [17] and are characterized by symptoms such as vomiting, nausea, dizziness, headaches, and more severe effects that can lead to death. The side effects of nicotine inhalation include headache, nausea, mouth/throat pain, cough, and heartburn [143]. Some users in our study may have been weaning themselves off nicotine or using devices with poor nicotine delivery leading to nicotine withdrawal, which could produce symptoms such as dizziness and anxiety [144]. Nicotine can trigger a dose-dependent loss of the endothelial barrier which has been shown to rapidly increase lung inflammation and oxidative stress in mice [15]. Nicotine in EC aerosols can induce glucose deprivation in the brain, which could lead to enhanced ischemic brain injury and or stroke risk. In addition, EC refill fluids may contain free-base nicotine (a form more addictive) [145], which can lead to greater deposition in the mouth and throat and upper respiratory tract [146].

Recently, an e-cigarette, or vaping, product use associated lung injury (EVALI) epidemic has been identified by the Centers for Disease Control and Prevention (CDC) [147]. As of December 2019, at least 2409 cases of lung injury have been reported to the CDC from 50 states, the District of Columbia, and 2 US territories [147]. In addition, 52 deaths associated with vaping were confirmed by 26 states and the District of Columbia [147]. Some of the commonly reported symptoms in presenting patients included chest pain, shortness of breath, cough, nausea, vomiting, diarrhea, fever, chills, fatigue/malaise, and headache [148-150], all of which are reported in this study. In addition to lung-related disease, some case reports included neurological and gastrointestinal symptoms [148,150], which overlap those found in our study.

The sudden uptick in health-related symptoms and conditions related to vaping comes at least 10 years after the products have gained widespread popularity in the United States, including the rise in popularity of JUUL and marijuana (THC) vape products. Our data show that many of the symptoms characterizing the current patients have been reported online for at least 7 years, suggesting that cases similar to those in the current epidemic have existed previously and been unreported or not linked to vaping. Our data further suggest that this epidemic will continue to grow given the many reports of symptoms characteristic of EVALI on the internet. The specific causes of the reported health effects are not yet known, but it is important to continue vigilant reporting of cases, tracking symptoms, and ongoing research on the health effects related to EC use to understand and contain the vaping epidemic.

### Limitations

Our data may underestimate positive health effects, which EC users are less likely to post on online forums. The factors causing the symptoms and disorders reported by EC users could be complex and will require further investigations. Demographic data on the study population were not extractable. It is not known if any individuals were dual users or if they had preexisting health conditions that may have affected their response to EC.

### Conclusions

This study is the first to use automated methods to analyze posts on an EC website over a span of 7 years and to identify the symptoms and disorders most frequently reported online by EC users. We demonstrate the value of using automated methods to acquire and analyze large datasets thereby increasing the power of infodemiological analyses. In addition, from our dataset, we identified a condensed list of symptoms and disorders and ranked them according to post frequency. These symptoms and disorders reported in our study may be of interest to physicians and health care providers who are treating patients using EC and could potentially be reported more frequently by EC users. Moreover, informative data were collected from a large population of EC vapers irrespective of their EC products and individual topographies and was not limited to a small selection of EC products or human subjects, as is often the case with experimental studies and case reports. Data collected using our automated method contribute to the growing body of knowledge linking EC use to adverse health effects, mainly in the mouth and throat and the neurological, respiratory, digestive, and integumentary systems. Our study identified hundreds of negative effects that were not previously described in case reports and peer-reviewed literature. The results from our study are in good agreement with previous surveys, human studies, and case reports. Although many of the symptoms that were reported with high frequency are not life-threatening (eg, headache, coughing, heartburn, sore throat), they can be disabling and reduce the quality of life. Of particular concern are the respiratory disorders that appeared with high frequency, such as asthma, COPD, pneumonia, and bronchitis, which not only severely impact the quality of life but may also be life threatening. Our data support the idea that EC use is not free of adverse health effects and that it is important to continue tracking the health of EC users. Advances in internet data mining provide a novel method for monitoring the health of EC users over time. Infodemiological data gathered on EC users will be valuable to physicians, regulatory agencies, and the users themselves.

## Appendix

Multimedia Appendix 1 Supplementary Figure 1: Heat map of symptoms less frequently reported in forums along with their associated systems. The total number of posts for each symptom is shown on a log scale.

Multimedia Appendix 2 Supplementary Figure 2: Heat map of disorders less frequently reported in forums along with their associated systems. The total number of posts for each disorder is shown on a log scale.

Multimedia Appendix 3 Supplementary Table 1: Listing of all symptoms not included in heat map.

Multimedia Appendix 4 Supplementary Table 2: Listing of all linked symptoms by frequency.

## Abbreviations

CDC: Centers for Disease Control and Prevention
COPD: chronic obstructive pulmonary disorder
CTP: Center for Tobacco Products
EC: electronic cigarette
FDA: Food and Drug Administration
PATH: Population Assessment of Tobacco Health
UMLS: Unified Medical Language System
VAPI: vaping-associated pulmonary illness
