# Supplementary figures and images for: Health Effects Associated With Electronic Cigarette Use: Automated Mining of Online Forums
Source: J Med Internet Res. 2020 Jan 3;22(1):e15684. doi: 10.2196/15684 (PMC6969389; doi:10.2196/15684)

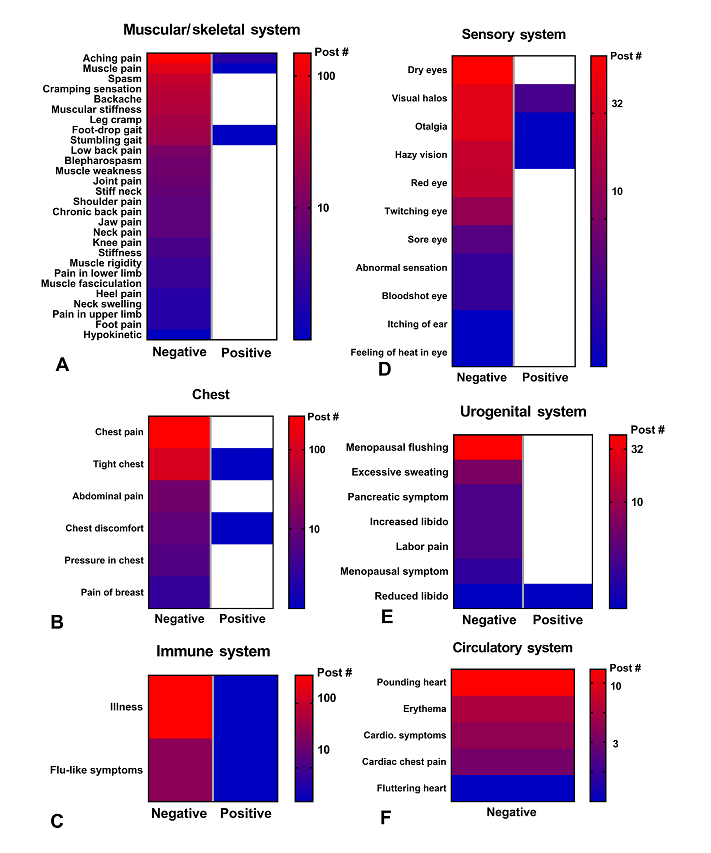

Supplement: Multimedia Appendix 1 [file jmir_v22i1e15684_app1.png]

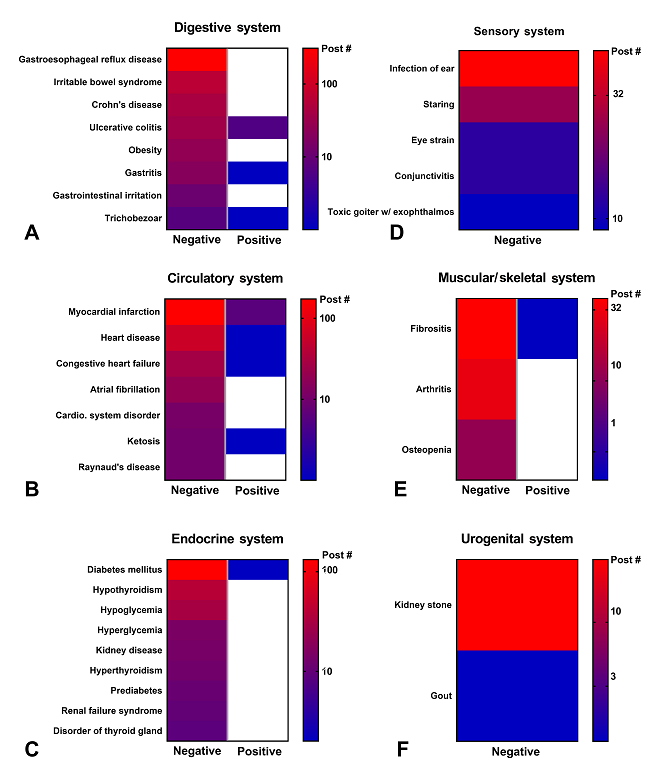

Supplement: Multimedia Appendix 2 [file jmir_v22i1e15684_app2.png]
