# Supplementary material for: Health Effects Associated With Electronic Cigarette Use: Automated Mining of Online Forums
Source: J Med Internet Res. 2020 Jan 3;22(1):e15684. doi: 10.2196/15684 (PMC6969389; doi:10.2196/15684)
Supplement: Multimedia Appendix 3 [file jmir_v22i1e15684_app3.docx]

Table 1: Listing of all symptoms not included in heat map

| System/Anatomical Region | Symptoms | Negative | Positive |
| --- | --- | --- | --- |
| Neurological System | Ocular headache | 1 (0.00028%) | 0 (0.00%) |
|  | Psychological symptom | 1 (0.00028%) | 0 (0.00%) |
|  | Spasmodic movement | 1 (0.00028%) | 0 (0.00%) |
|  | Death anxiety | 1 (0.00028%) | 0 (0.00%) |
|  | Feeling feverish | 1 (0.00028%) | 0 (0.00%) |
|  | Restless sleep | 1 (0.00028%) | 0 (0.00%) |
|  | Incoordination | 1 (0.00028%) | 0 (0.00%) |
|  | Partial seizure | 1 (0.00028%) | 0 (0.00%) |
|  | Death rattle | 0 (0.00%) | 1 (0.00028%) |
|  | Peripheral neuropathic pain | 1 (0.00028%) | 0 (0.00%) |
|  | Felt faint | 1 (0.00028%) | 0 (0.00%) |
|  | Increased nervousness | 1 (0.00028%) | 0 (0.00%) |
|  | Increased thirst | 1 (0.00028%) | 0 (0.00%) |
| System/Anatomical Region | Symptoms | Negative | Positive |
| Respiratory System | Whopping respiration | 1 (0.0005%) | 0 (0.00%) |
|  | Early morning cough | 0 (0.00%) | 0 (0.00%) |
|  | Grunting respiration | 1 (0.0005%) | 0 (0.00%) |
|  | Catarrh | 0 (0.00%) | 0 (0.00%) |
|  | Interrupted breathing | 0 (0.00%) | 0 (0.00%) |
|  | Hemoptysis | 1 (0.0005%) | 0 (0.00%) |
|  | Paroxysmal nocturnal dyspnea | 1 (0.0005%) | 0 (0.00%) |
|  | Respiration intermittent | 1 (0.0005%) | 0 (0.00%) |
|  | Labored breathing | 1 (0.0005%) | 0 (0.00%) |
|  | Chest pain on breathing | 1 (0.0005%) | 0 (0.00%) |
| System/Anatomical Region | Symptoms | Negative | Positive |
| Digestive System | Nausea and vomiting | 1 (0.00061%) | 0 (0.00%) |
|  | Abdominal discomfort | 1 (0.00061%) | 0 (0.00%) |
|  | Hematemesis | 1 (0.00061%) | 0 (0.00%) |
|  | Weight symptom | 1 (0.00061%) | 0 (0.00%) |
|  | Infantile diarrhea | 1 (0.00061%) | 0 (0.00%) |
|  | Abdominal tenderness | 1 (0.00061%) | 0 (0.00%) |
|  | Morning sickness | 0 (0.00%) | 0 (0.00%) |
|  | Morning nausea | 0 (0.00%) | 0 (0.00%) |
|  | Severe diarrhea | 1 (0.00061%) | 0 (0.00%) |
|  | Digestive symptom | 1 (0.00061%) | 0 (0.00%) |
|  | Flatulent dyspepsia | 1 (0.00061%) | 0 (0.00%) |
| System/Anatomical Region | Symptoms | Negative | Positive |
| Mouth and Throat | Oral hypoesthesia | 1 (0.00072%) | 0 (0.00%) |
|  | Sore lip | 1 (0.00072%) | 0 (0.00%) |
|  | Excessive thirst | 1 (0.00072%) | 0 (0.00%) |
|  | Change in voice | 1 (0.00072%) | 0 (0.00%) |
|  | Garlic breath | 1 (0.00072%) | 0 (0.00%) |
| System/Anatomical Region | Symptoms | Negative | Positive |
| Integumentary System | Warm skin | 1 (0.0012%) | 0 (0.00%) |
|  | Lamellar nail splitting | 1 (0.0012%) | 0 (0.00%) |
|  | Cushingoid facies | 1 (0.0012%) | 0 (0.00%) |
|  | Face acne | 1 (0.0012%) | 0 (0.00%) |
|  | Thick skin | 1 (0.0012%) | 0 (0.00%) |
| System/Anatomical Region | Symptoms | Negative | Positive |
| Muscular/Skeletal System | Hypokinetic | 1 (0.0016%) | 0 (0.00%) |
|  | Pain in wrist | 1 (0.0016%) | 0 (0.00%) |
|  | Hip pain | 0 (0.00%) | 0 (0.00%) |
|  | Charleyhorse | 1 (0.0016%) | 0 (0.00%) |
|  | Tenalgia | 1 (0.0016%) | 0 (0.00%) |
|  | Pain in finger | 1 (0.0016%) | 0 (0.00%) |
|  | Myokymia | 1 (0.0016%) | 0 (0.00%) |
|  | Mechanical pain | 1 (0.0016%) | 0 (0.00%) |
|  | Involuntary movement symptom | 1 (0.0016%) | 0 (0.00%) |
|  | Joint stiffness | 1 (0.0016%) | 0 (0.00%) |
| System/Anatomical Region | Symptoms | Negative | Positive |
| Chest | Intolerant of cold |  |  |
|  | Swallowing painful |  |  |
|  | Atypical chest pain |  |  |
|  | Radiating chest pain |  |  |
|  | Right sided chest pain |  |  |
| System/Anatomical Region | Symptoms | Negative | Positive |
| Immune System | Intolerant of cold | 1 (0.0028%) | 0 (0.00%) |
| System/Anatomical Region | Symptoms | Negative | Positive |
| Sensory System | Auditory hallucinations | 1 (0.0038%) | 0 (0.00%) |
|  | Photophobia | 1 (0.0038%) | 0 (0.00%) |
|  | Loss of sense of smell | 1 (0.0038%) | 0 (0.00%) |
|  | Photopsia | 1 (0.0038%) | 0 (0.00%) |
|  | Ear symptom | 1 (0.0038%) | 0 (0.00%) |
|  | Discharge from eye | 1 (0.0038%) | 0 (0.00%) |
|  | Rolling of eyes | 1 (0.0038%) | 0 (0.00%) |
|  | Eye swelling | 1 (0.0038%) | 0 (0.00%) |
| System/Anatomical Region | Symptoms | Negative | Positive |
| Urogenital System | Polyuria | 1 (0.014%) | 0 (0.00%) |
|  | Menopausal problem | 1 (0.014%) | 0 (0.00%) |
| System/Anatomical Region | Symptoms | Negative | Positive |
| Circulatory System | Abnormal heart beat | 1 (0.02%) | 0 (0.00%) |
|  | Widened pulse pressure | 1 (0.02%) | 0 (0.00%) |
