# Supplementary material for: Health Effects Associated With Electronic Cigarette Use: Automated Mining of Online Forums
Source: J Med Internet Res. 2020 Jan 3;22(1):e15684. doi: 10.2196/15684 (PMC6969389; doi:10.2196/15684)
Supplement: Multimedia Appendix 4 [file jmir_v22i1e15684_app4.docx]

Table 2: Listing of all linked symptoms by frequency

| First Symptom | Second Symptom | Frequency |
| --- | --- | --- |
| Nausea | Headache | 76 |
| Wheezing | Coughing | 72 |
| Pain in throat | Coughing | 69 |
| Pain in throat | Headache | 62 |
| Coughing | Headache | 42 |
| Headache | Itching | 29 |
| Tired | Headache | 28 |
| Dizziness | Headache | 28 |
| Aching pain | Headache | 27 |
| Vomiting | Nausea | 27 |
| Malaise | Headache | 26 |
| Pain in throat | Nasal discharge | 26 |
| Headache | Fatigue | 25 |
| Pharyngeal dryness | Pain in throat | 22 |
| Coughing | Nasal discharge | 22 |
| Headache | Nasal discharge | 22 |
| Malaise | Illness | 21 |
| Dry skin | Itching | 21 |
| Lightheadedness | Headache | 20 |
| Headache | Cramp | 20 |
| Tired | Fatigue | 19 |
| Malaise | Coughing | 19 |
| Aching pain | Coughing | 18 |
| Pharyngeal dryness | Headache | 18 |
| Nausea | Itching | 18 |
| Coughing | Fatigue | 18 |
| Headache | Muscle pain | 18 |
| Vomiting | Headache | 17 |
| Chest pain | Headache | 17 |
| Indigestion | Heartburn | 16 |
| Wheezing | Dyspnea | 16 |
| Nausea | Cramp | 16 |
| Deep breathing | Coughing | 16 |
| Headache | Constipation | 16 |
| Lightheadedness | Nausea | 15 |
| Upset stomach | Headache | 15 |
| Pain in throat | Itching | 15 |
| Insomnia | Headache | 15 |
| Aching pain | Pain in throat | 14 |
| Numbness | Tingling sensation | 14 |
| Diarrhea | Vomiting | 14 |
| Upset stomach | Nausea | 14 |
| Wheezing | Headache | 14 |
| Pharyngeal dryness | Coughing | 14 |
| Pain in throat | Tight chest | 14 |
| Pain in throat | Nausea | 14 |
| Pain in throat | Fatigue | 14 |
| Nausea | Coughing | 14 |
| Nausea | Dizziness | 14 |
| Illness | Headache | 14 |
| Tired | Nausea | 13 |
| Diarrhea | Headache | 13 |
| Withdrawal symptom | Headache | 13 |
| Malaise | Nausea | 13 |
| Wheezing | Tight chest | 13 |
| Nausea | Dyspnea | 13 |
| Chest pain | Coughing | 13 |
| Illness | Coughing | 13 |
| Coughing | Itching | 13 |
| Nasal congestion | Pain in throat | 12 |
| Malaise | Pain in throat | 12 |
| Nausea | Constipation | 12 |
| Tight chest | Coughing | 12 |
| Heartburn | Headache | 12 |
| Headache | Hangover | 12 |
| Aching pain | Muscle pain | 11 |
| Lightheadedness | Dizziness | 11 |
| Dry cough | Coughing | 11 |
| Diarrhea | Nausea | 11 |
| Pain in throat | Illness | 11 |
| Nausea | Fatigue | 11 |
| Chest pain | Heartburn | 11 |
| Coughing | Dyspnea | 11 |
| Headache | Chill | 11 |
| Muscle pain | Cramp | 11 |
| Dry cough | Pain in throat | 10 |
| Nasal congestion | Headache | 10 |
| Harsh voice quality | Pain in throat | 10 |
| Wheezing | Pain in throat | 10 |
| Wheezing | Itching | 10 |
| Pain in throat | Heartburn | 10 |
| Pain in throat | Constipation | 10 |
| Abdominal bloating | Constipation | 10 |
| Tight chest | Headache | 10 |
| Heartburn | Coughing | 10 |
| Dyspnea | Itching | 10 |
